# Stem cell–based therapies for inherited retinal diseases – Translational advances and clinical evidence: A review

**DOI:** 10.17305/bb.2026.13483

**Published:** 2026-01-22

**Authors:** Yuwei Huang, Yuan Xie, Chongru Wang

**Affiliations:** 1 Department of Ophthalmology, Renmin Hospital, Hubei University of Medicine, Shiyan, Hubei, China

**Keywords:** Cell replacement, gene editing, immunomodulation, retinal degeneration, retinal pigment epithelium

## Abstract

Inherited retinal diseases (IRDs) represent a genetically diverse group of disorders that result in the progressive degeneration of photoreceptors and/or retinal pigment epithelium (RPE), ultimately leading to significant vision loss and diminished quality of life. Symptoms vary widely, encompassing night blindness, peripheral vision loss, central vision impairment, and total blindness, with disease progression influenced by the specific genetic mutation and inheritance pattern. This narrative review synthesizes recent findings on the pathogenesis of IRDs and examines stem cell-based interventions across preclinical models and early clinical trials. Mutations in genes such as *RPE65*, *ABCA4*, and *USH2A* disrupt critical retinal pathways, contributing to oxidative stress, inflammation, and apoptosis. Stem cell strategies, including pluripotent stem cell-derived RPE/photoreceptor precursors, mesenchymal stem cells, and retinal progenitor cells, offer potential mechanisms for limited cellular replacement and synaptic integration, as well as paracrine neuroprotection and immunomodulation. Current research indicates feasible delivery methods (intravitreal, subretinal, or suprachoroidal) with generally acceptable safety profiles; however, functional improvements in vision are often inconsistent and temporary, and durable vision restoration remains unproven. Significant challenges persist, including immune rejection, tumorigenicity risks, weak engraftment, technical complexity, and regulatory barriers. These issues underscore the necessity for standardized manufacturing processes and well-controlled, long-term clinical trials to advance the field of IRD treatment.

## Introduction

Inherited retinal diseases (IRDs) lead to progressive degeneration of photoreceptors or the retinal pigment epithelium (RPE), culminating in vision loss. Affecting approximately 1 in 4,000 individuals globally, retinitis pigmentosa (RP) is the most prevalent subtype [1, 2]. Currently, around 300 genes associated with IRDs have been identified, highlighting the genetic diversity of these disorders. The impact of IRDs on an individual’s quality of life (QoL) is profound, resulting in progressive vision loss that imposes social, psychological, and financial constraints [1]. These challenges lead to diminished independence, reduced employment opportunities, and disruption of daily routines. More than 270 genes have been documented as contributing to the pathophysiology of IRDs, leading to structural and functional alterations within the retina that complicate disease progression [2]. The inheritance patterns of IRDs—X-linked, autosomal dominant, or autosomal recessive—carry specific implications for family planning, genetic counseling, and diagnosis [2, 3]. Due to the genetic diversity and individual variations in disease onset, accurate diagnosis and tailored treatment plans pose significant challenges. Rod photoreceptor degeneration characterizes RP, initially impairing night and peripheral vision, followed by central vision loss and eventual blindness [1]. Leber congenital amaurosis (LCA), a severe IRD, manifests in infancy and leads to blindness or early-onset visual impairment. Stargardt disease (STGD) predominantly affects the macula, resulting in gradual central vision loss [1, 2]. This review elucidates recent studies and evidence regarding stem cell-based interventions for IRDs, detailing their pathogenesis, disease progression, therapeutic mechanisms, and investigational protocols.

### Molecular pathogenesis of IRDs

**Figure 1.**
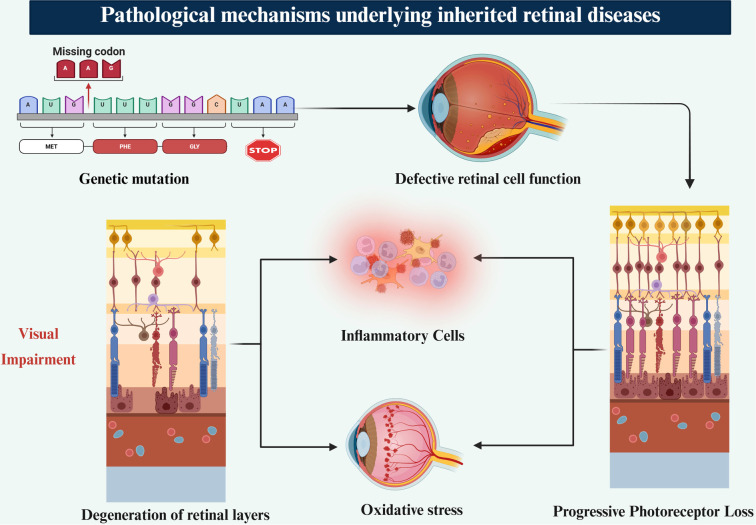
**Pathophysiology of IRDs.** Schematic overview of the disease cascade in IRDs. Primary genetic mutations impair photoreceptor and/or RPE function, promoting the accumulation of toxic by-products. These disturbances trigger OS and activation of inflammatory cells, which amplify tissue injury through a self-propagating feedback loop. The resulting milieu accelerates progressive photoreceptor loss, degeneration of retinal layers, and ultimately visual impairment. Abbreviations: IRDs: Inherited retinal diseases; RPE: Retinal pigment epithelium; OS: Oxidative stress.

The molecular pathogenesis of IRDs encompasses a broad spectrum of gene mutations that disrupt retinal cell function and survival. Mutations in genes such as RPE65, ABCA4, and USH2A compromise critical biological pathways, including the visual cycle, photoreceptor renewal, and cellular structural maintenance [1, 2]. RPE65 mutations impair the conversion of all-trans-retinyl esters to 11-cis-retinol, a pivotal step in phototransduction, resulting in photoreceptor cell death [1, 4]. ABCA4 mutations obstruct the clearance of toxic bisretinoids in the RPE, leading to oxidative stress and lipofuscin accumulation (Figure 1) [2, 5, 6]. These molecular insults instigate inflammation, mitochondrial dysfunction, and apoptotic cascades, particularly affecting the RPE and photoreceptors [3, 7, 8]. Mutations in USH2A are linked to Usher syndrome, where defective extracellular matrix (ECM) proteins contribute to dual sensory loss affecting both the retina and cochlea [2, 7, 9]. Disrupted proteostasis, impaired autophagy, and glial activation exacerbate retinal degeneration [4, 10]. Optical coherence tomography (OCT) typically reveals thinning of the outer nuclear layer and RPE, aligning with disease progression [4]. A comprehensive understanding of these mechanisms is crucial for developing gene and cell-based interventions, facilitating more precise, mutation-specific therapeutic strategies [6, 8, 11].

### Clinical progression and manifestations of IRDs

The visual symptoms of IRDs vary widely, ranging from mild night blindness to complete vision loss, with severity and progression influenced by genetic mutations. Early indications often include nyctalopia, slow dark adaptation, and peripheral field defects [8]. As the disease advances, patients may experience tunnel vision, loss of color discrimination, and primary vision loss, significantly impacting daily functioning and overall quality of life [12]. Classic ophthalmoscopic features of RP include bone spicule pigmentation, attenuation of retinal vessels, and waxy pallor of the optic disc [13]. The rate of degeneration can vary significantly, even among family members, underscoring the genetic heterogeneity and phenotypic variability of IRDs [8]. Some subtypes, such as Usher syndrome, are characterized by syndromic features like sensorineural hearing loss, complicating diagnosis and management [12]. Therefore, accurate diagnosis and disease staging through multimodal imaging and genetic testing are essential for determining prognosis and therapeutic approaches, particularly as gene and cell-based interventions emerge [8, 12, 13].

### Stem cells in retinal therapy

Stem cell-based therapies aim to address degenerative retinal disorders, including RP and other IRDs. Research has explored the potential of embryonic, induced pluripotent, mesenchymal, and retinal progenitor cells (RPCs) to restore retinal structure and function.

#### Embryonic stem cells (ESCs)

ESCs, derived from the inner cell mass of the blastocyst, possess pluripotent capabilities, allowing differentiation into photoreceptors and RPE cells. Various methods have been developed to drive ESCs toward RPE differentiation, essential for maintaining retinal homeostasis and supporting photoreceptor survival, establishing them as a primary source for retinal regeneration [6, 14, 15]. ESC-derived RPE cells have demonstrated structural integration and sustained survival, enhancing visual acuity across numerous studies and clinical trials. However, challenges such as tumorigenicity, ethical concerns regarding embryo use, and the risk of immunological rejection necessitate specific immunosuppressive treatments, limiting the widespread application of ESC-derived therapies [6, 9, 16, 17].

#### Induced pluripotent stem cells (iPSCs)

iPSCs are generated by reprogramming adult somatic cells, such as skin fibroblasts, into a pluripotent state. This technique minimizes the risk of immunological rejection and avoids ethical dilemmas associated with embryonic sources [6, 18, 19]. iPSCs can differentiate into photoreceptors, RPE cells, and other retinal cell types, supporting their application in tissue engineering, drug screening, and disease modeling [6, 14, 16, 18]. Preliminary clinical studies employing iPSC-derived RPE sheets for RP have demonstrated feasibility and safety [6, 12, 19].

#### Mesenchymal stem cells (MSCs)

MSCs, derived from sources such as bone marrow, adipose tissue, and umbilical cord, exhibit paracrine and immunomodulatory properties that contribute to retinal preservation. They produce various neurotrophic factors that promote tissue repair, reduce inflammation, and enhance the survival of retinal cells [20–24]. Numerous early-phase clinical trials in RP and optic neuropathies have validated the safety of MSCs, revealing modest improvements in visual function, despite their limited differentiation into retinal-specific cells [21, 23, 25]. Their low structural integration and restricted specificity for retinal lineages suggest that the therapeutic effects of MSCs are predominantly mediated through trophic support rather than direct cellular replacement [16, 20, 21].

#### RPCs

Retinal progenitor cells, which arise during retinal development, can differentiate into retinal neurons, including photoreceptors and interneurons [7, 9, 16, 24, 26]. Given their developmental commitment to retinal lineages, RPCs offer a more targeted approach for retinal cell replacement, with a lower risk of tumor formation compared to pluripotent stem cells. Preclinical studies and early human trials have indicated that transplanted RPCs can survive, migrate, and integrate into the degenerating retina, partially restoring visual function. These cells have been the subject of clinical trials targeting inherited retinal dystrophies [7, 13, 16, 26]. ESC- and iPSC-derived RPE cells have demonstrated sustained survival and functional improvement in these models [6, 15, 19]. MSCs exhibit neuroprotective effects in RP and diabetic retinopathy through anti-inflammatory and paracrine actions [21, 23, 27]. Both RPCs and neural stem cells show promise in photoreceptor rescue and visual function restoration in preclinical and clinical settings [7, 13, 26]. Emerging strategies, including stem cell-derived secretomes, biodegradable scaffolds, and gene correction technologies, aim to enhance therapeutic outcomes [27–30], thereby improving clinical applications. Investigations into the intravitreal injection of autologous bone marrow or mesenchymal stem cells for RP and IRDs have been conducted, although variable outcomes and complications, such as epiretinal membrane formation, have been noted [31–36]. Transplantation of stem cell-derived RPE sheets, either using scaffolds or as monolayers, has yielded promising results in STGD, with improved survival and partial restoration of vision [17, 19, 25]. The combination of gene correction with stem cell therapy, particularly utilizing iPSCs, offers a targeted approach for genetic retinal diseases such as RP, choroideremia, and Stargardt disease [12, 13, 19, 28]. Advances in delivery systems and biomaterials have enhanced cell survival, integration, and therapeutic efficacy in retinal regenerative medicine.

### Current management challenges and the promise of stem cell therapies for IRDs

Currently, there are limited therapeutic options for IRDs, with no widely accepted curative therapies available. Although gene therapy and retinal prostheses can restore some vision or delay disease progression, they are often mutation-specific and most effective in the early stages of the disease. Typically, by the time of diagnosis, photoreceptors and RPE cells experience significant irreparable damage, complicating treatment and reducing therapeutic efficacy [13]. Despite advancements in assistive technologies, many patients experience functional blindness, particularly in central and night vision [10]. Consequently, stem-cell-based therapies hold considerable promise for slowing disease progression, replacing lost retinal cells, and differentiating into RPE and ganglion cells [8, 9]. Innovations such as gene editing and personalized medicine have enhanced the therapeutic potential of stem cell modalities [11]. Targeted treatments can address specific genetic mutations, improving efficacy while minimizing risks; however, challenges related to safety and accessibility remain [5, 6]. Emerging technologies, including paracrine and secretome-based therapies, offer neuroprotective and anti-inflammatory benefits in conditions such as RP and glaucoma [27, 29, 33]. Preliminary results from multiple phase I/II trials have indicated safety and efficacy [11, 37–39], but challenges such as immune rejection, tumorigenicity, and regulatory complexities hinder widespread application [16, 37, 40–42].

### Scaffold-based approaches for retinal cell transplantation

A significant limitation of stem cell therapy for IRDs is the poor survival and inconsistent integration of transplanted cells when administered as suspensions. Biodegradable scaffolds designed to mimic Bruch’s membrane provide a stable surface for organized RPE monolayers, enhancing graft retention and directional trophic support following subretinal placement [17]. Parylene and gelatin-based matrices offer superior photoreceptor preservation compared to free-cell suspensions [6]. However, the use of scaffolds increases surgical complexity and may provoke inflammatory reactions due to material degradation [17]. While cell suspensions enable less invasive delivery via intravitreal or suprachoroidal routes, they often result in inadequate engraftment, cell clustering, and insufficient functional recovery [6]. Thus, scaffold-supported delivery presents a more organized approach for retinal repair.

### Mechanisms of stem cell action in retinal diseases

**Figure 2.**
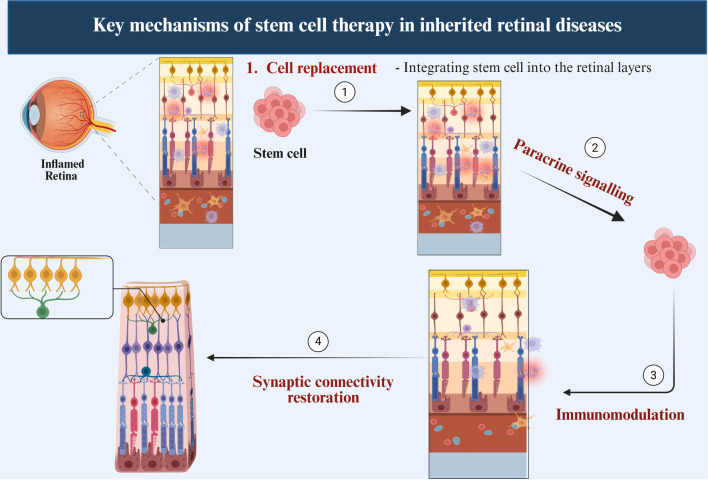
**Mechanisms of stem cell action in IRDs.** Schematic representation of the principal, interconnected pathways through which SC therapies may promote retinal repair in IRDs. (1) Cell replacement: Transplanted SCs engraft within degenerated retinal layers and differentiate into retinal lineages—most notably PRs and/or RPE—to replenish lost cells. (2) Paracrine signalling: SCs release neurotrophic and cytoprotective mediators (e.g., BDNF, CNTF, GDNF, PEDF) that enhance host-cell survival, stabilize the retinal microenvironment, and attenuate OS. (3) Immunomodulation: SCs reduce chronic retinal inflammation by suppressing pro-inflammatory pathways, limiting microglial activation, and promoting anti-inflammatory cytokine signalling (e.g., IL-10, TGF-β). (4) Synaptic connectivity restoration: Graft-derived PRs mature and establish synaptic contacts with host bipolar and horizontal cells, supporting reconstitution of disrupted retinal circuitry. The relative contribution of these mechanisms depends on the SC source (e.g., hESC-/iPSC-derived retinal cells, MSCs, RPCs), disease stage, and delivery context. Abbreviations: IRDs: Inherited retinal diseases; SC: Stem cell; PRs: Photoreceptors; RPE: Retinal pigment epithelium; BDNF: Brain-derived neurotrophic factor; CNTF: Ciliary neurotrophic factor; GDNF: Glial cell line-derived neurotrophic factor; PEDF: Pigment epithelium-derived factor; OS: Oxidative stress; IL-10: Interleukin-10; TGF-β: Transforming growth factor-beta; hESC: Human embryonic stem cell; iPSC: Induced pluripotent stem cell; MSCs: Mesenchymal stem cells; RPCs: Retinal progenitor cells.

Stem cell-based therapies operate through multiple interconnected mechanisms, primarily involving cell replacement, paracrine signaling, immunomodulation, and the restoration of synaptic connectivity (Figure 2). These mechanisms contribute to slowing disease progression and may restore visual function in patients with retinal degeneration. In retinal stem cell therapy, transplanted stem cells replace lost photoreceptors and RPE cells by integrating into degenerated retinal layers. Preclinical and early-phase clinical studies have demonstrated that human embryonic stem cell (hESC)-derived RPE cells can survive, migrate, and integrate into the subretinal space, exhibiting both morphological and functional characteristics of native RPE cells [31, 40, 43]. Photoreceptor precursors derived from iPSCs or RPCs have successfully incorporated into the outer nuclear layer and expressed mature photoreceptor markers [20, 34]. These findings underscore the potential of stem cell therapy to restore retinal structure and function in dystrophies.

Stem cells also provide paracrine and trophic support by releasing neuroprotective factors that promote retinal cell survival and mitigate degeneration. MSCs secrete key cytokines and growth factors, including brain-derived neurotrophic factor (BDNF), ciliary neurotrophic factor (CNTF), glial cell line-derived neurotrophic factor (GDNF), and pigment epithelium-derived factor (PEDF) [5, 6, 26]. These molecules help maintain retinal structure, protect host photoreceptors, and modulate the surrounding microenvironment. In models of retinal degeneration, these paracrine effects have been associated with delayed photoreceptor loss and improved retinal function, as evidenced by enhanced electroretinogram (ERG) responses [32, 44].

Additionally, MSCs possess immunomodulatory properties that are critical for retinal therapies. They help suppress chronic retinal inflammation, a common feature of degenerative and autoimmune retinal diseases. MSCs secrete anti-inflammatory cytokines, such as interleukin-10 (IL-10) and transforming growth factor-beta (TGF-β), which inhibit pro-inflammatory responses and promote immune tolerance [15, 26]. Moreover, stem cells can reduce microglial activation and restrict the infiltration of immune cells into retinal tissue, thereby preventing further immune-mediated neuronal damage [45]. This aspect is particularly relevant in autoimmune uveitis and RP, where inflammation exacerbates photoreceptor loss.

The restoration of synaptic connectivity is another crucial mechanism. For stem cell therapies to be functionally effective, grafted cells must survive, integrate, and establish appropriate synaptic connections with existing retinal neurons. Experimental models have shown that transplanted photoreceptors can form synaptic structures with host bipolar and horizontal cells, indicating the potential for re-establishing disrupted visual circuits [20, 43]. Although complete functional restoration remains a challenge, advancements in stem cell differentiation protocols and transplantation techniques are enhancing the efficacy of synaptic integration. Future success will depend on refining these mechanisms, ensuring safety, and improving delivery methods to achieve effective clinical translation and vision restoration.

### Routes of stem cell delivery in retinal diseases

Various delivery techniques have been explored, each tailored to the target retinal layer and specific disease pathology. These techniques possess distinct advantages and disadvantages concerning cell survival, integration, surgical feasibility, and potential complications. Table 1 presents a comparative overview of the primary delivery approaches utilized in clinical and preclinical settings.

**Table 1:** Routes of stem cell delivery for retinal regeneration: Key features, advantages, challenges, and current status

**Routes**	**Chief features**	**Advantages**	**Challenges**	**Current status**
**Intravitreal injection**	Cells injected into the vitreous cavity. Targets ganglion cell layer and inner retina.	Minimally invasive, repeatable, safe technique [27, 46].	Limited cell migration to outer retina; risk of inflammation or ERM [35].	Widely used in trials; focus on neuroprotection [13, 27, 40].
**Subretinal injection**	Cells delivered between neurosensory retina and RPE. Directly targets photoreceptors and RPE.	Precise delivery; promotes photoreceptor integration [6, 9, 16].	Technically demanding; retinal detachment risk; limited spread [16].	Most effective for vision restoration in RPE/photoreceptor loss [6, 7].
**Suprachoroidal injection**	Injection into the potential space between sclera and choroid. Targets choroid/RPE interface.	Less invasive than subretinal; wide diffusion [8, 22].	Limited human data; cell homing to retina uncertain [16, 22].	Promising preclinical and early clinical data [42].
**Subtenon and epiretinal**	Subtenon: under Tenon’s capsule; Epiretinal: on retinal surface (ILM side).	Experimental routes; potential slow-release delivery [18].	Inconsistent targeting; limited cell integration; under evaluation [18, 35].	Preclinical; not yet standard; being explored in select studies [18, 35, 47].

Abbreviations: BCVA: Best-corrected visual acuity; ERM: Epiretinal membrane; ILM: Internal limiting membrane; RPE: Retinal pigment epithelium.

**Table 2:** Summary of clinical trials on stem cell-based therapies for IRDs

**Author/year**	**Disease**	**Stem cell type and dose**	**Delivery**	**Registry/phase**	**Sample (treated)**	**Key outcomes**	**Safety**
Zhao et al. (2020) [48]	RP	UC-MSCs; 1×10^8^ cells	IV	ChiCTR-ONC-16008839; I/II	32 pts (64 eyes)	BCVA gain was defined as ≥5 letters. At 12 months, 81.3% of patients maintained or improved BCVA. NEI-VFQ25 scores rose at 3 months. Visual field sensitivity and FVEP showed no change.	No SAEs; no tumorigenesis, rejection, or vascular leakage.
Weiss et al. (2018) (SCOTS/SCOTS2) [36]	RP	Autologous BMSC (∼1.2B total nucleated cells)	Retrobulbar + Subtenon ± Intravitreal + IV	NCT01920867/ NCT03011541	17 pts (33 eyes)	Improvement was defined as a ≥1-line Snellen gain. Overall, 45.5% of eyes improved, 45.5% remained stable, and 9% worsened. Mean gain was 7.9 Snellen lines, with a 31% LogMAR improvement (*P* ═ 0.016).	No reported surgical or inflammatory complications.
Mehat et al. (2018) [25]	STGD	hESC-RPE (MA09-hRPE); 50,000–200,000 cells	Subretinal	NCT01469832; I/II	12 pts (12 eyes)	All participants showed hyperpigmentation, indicating graft survival. No one achieved functional improvement: BCVA changed ≤5 ETDRS letters, and microperimetry sensitivity showed no significant gains at 12 months. One high-dose case had localized retinal thinning with reduced sensitivity.	No cell-related SAEs; procedure-linked events: retinal dialysis (1), subretinal hemorrhage (2), pigment dispersion (4); immunosuppression AEs in 5.
Tuekprakhon et al. (2021) [51]	RP	Autologous BM-MSCs (1×10^6^/5×10^6^/ 1 × 10^7^)	Intravitreal	NCT01531348; Phase I	14 pts	BCVA showed transient significant gains (–0.18 logMAR in 1×10^6^ group at months 7–8, *P* < 0.05) but returned to baseline by 12 months. No participants consistently met the ≥5 ETDRS letter threshold. VF and CST remained stable. Patient-reported outcomes: 50% stable vision, 35.7% improved dim-light tasks.	Mild transient inflammation; transient IOP spikes; single cases: synechiae, CME, choroidal detachment; long-term: 1 vitreous hemorrhage with osseous metaplasia (resolved).
Kahraman et al. (2020) [52]	RP	UC-MSCs; 5M cells/eye	Suprachoroidal (Limoli)	Turkish MoH 56733164/203; Phase III	124 eyes/82 pts	Mean BCVA improved 0.27 logMAR (1.36 → 1.09). Visual Field Mean Deviation (VF MD) improved 28.12 → 24.19 dB (P < 0.05), and central mfERG P1 amplitudes increased. Based on BCVA changes, 46% improved, 42% stable, 12% worsened.	No major ocular/systemic issues; 1 transient vision loss episode; temporary VF defect resolved.
Oner et al. (2025) [53]	RP	UC-MSCs; 5M cells/eye	Suprachoroidal	Turkish MoH 56733164/203; Phase III (long-term)	669 eyes/429 pts	BCVA improved 0.30 logMAR at 2 years and remained +0.17 logMAR at 4 years. VF MD increased +1.36 dB at 2 years (*P* < 0.05). Central mfERG P1 amplitudes rose significantly and persisted to 4 years. FST thresholds improved at 1 year (white stimulus +9.7 dB).	No therapy-related SAEs; common: conjunctival hyperemia (67%), light sensitivity (18%). Two myopic RD cases (not linked).
Özmert et al. (2020) [54]	RP	WJ-MSCs 2–6M cells; GMP P3	Sub-Tenon (deep)	SHGM56733164; Phase III	32 pts (34 eyes)	BCVA improved 10.1 ETDRS letters (70.5 → 80.6, *P* ═ 0.01). VF MD improved 2.6 dB (27.3 → 24.7, *P* ═ 0.01). Outer retinal thickness increased 100 → 119 µm (*P* ═ 0.01). Central mfERG P1 amplitude and implicit time improved (*P* ≤ 0.02); peripheral unchanged. Flicker ERG amplitude increased and implicit times decreased (*P* ═ 0.01).	No SAEs; no inflammation, IOP rise, rejection, RD; 1 transient nystagmus increase.
Liu et al. (2017) [55]	RP	Human fetal-derived RPCs; 1×10^6^ cells	Subretinal	ChiCTR-TNRC-08000193; Phase I	8 pts	BCVA improved 10.1 ETDRS letters (70.5 → 80.6, *P* ═ 0.01). VF MD increased 2.6 dB (27.3 → 24.7, *P* ═ 0.01). Outer retinal thickness rose 100 → 119 µm (*P* ═ 0.01). Central mfERG P1 amplitude and implicit time improved (*P* ≤ 0.02); peripheral unchanged. Flicker ERG amplitude increased, implicit times decreased (*P* ═ 0.01).	No rejection, tumors, RD, endophthalmitis, CME. One ERM at 12 months. Imaging remained stable.

Abbreviations: BCVA: Best-corrected visual acuity; VF: Visual field; FVEP: Flash visual evoked potential; NEI-VFQ25: National Eye Institute Visual Function Questionnaire-25; BMSC: Bone marrow–derived stem cells; hESC-RPE: Human embryonic stem cell–derived retinal pigment epithelium; CST: Central subfield thickness; mfERG: Multifocal electroretinography; FST: Full-field stimulus threshold; WJ-MSC: Wharton’s jelly–derived mesenchymal stem cells; ETDRS: Early Treatment Diabetic Retinopathy Study; RD: Retinal detachment; CME: Cystoid macular edema; RPCs: Retinal progenitor cells; SAEs: Serious adverse events; IOP: Intraocular pressure.

### Preclinical studies and animal models

Preclinical animal studies assess the safety and efficacy of retinal cell transplantation for IRDs. Rodent models, such as retinal degeneration 1 (rd1) and retinal degeneration 10 (rd10) mice, as well as non-human primates, effectively replicate the degenerative retinal changes characteristic of IRDs [14, 22, 39, 47, 48]. These models facilitate the investigation of donor cell behaviors, including survival, migration, and integration into the host retina. Genetically engineered models that replicate specific mutations associated with human IRDs enhance the translation of preclinical findings and support the development of targeted therapeutic approaches [14, 22, 24, 49]. Transplantation studies in these animal models have yielded positive outcomes, including restoration of visual acuity and functional improvements [22, 39, 48, 50]. Histological and molecular analyses have demonstrated donor cell survival, migration, and partial synaptic integration with host retinal circuits, thereby confirming functional integration [13, 39, 48]. Numerous preclinical studies have utilized well-differentiated human pluripotent stem cell-derived retinal cells, such as hESC- or iPSC-derived RPE or photoreceptor precursors, transplanted into immunodeficient or immunosuppressed rodent models (rd1 or rd10 mice) to evaluate safety and survival, with follow-up periods ranging from 6 to 12 months [14, 39, 49]. These grafts necessitated systemic immunosuppression, such as that provided by tacrolimus or cyclosporine, to prevent rejection, with no instances of teratoma formation reported [14, 49]. An alternative approach involving mesenchymal stem cells in immunocompetent models demonstrated minimal immune response and a lack of need for immunosuppression due to their low major histocompatibility complex (MHC) class II expression and immunomodulatory properties [20, 23]. These findings support short- to medium-term graft viability; however, challenges such as immune compatibility, retinal remodeling, and incomplete disease modeling persist [12, 24, 39]. Further refinement of models, graft preparation, delivery methods, and immunomodulation strategies is essential for clinical applications [14, 49].

### Clinical trials and translational progress

The clinical translation of retinal cell therapies for IRDs has progressed significantly, with key trials confirming both safety and potential efficacy. Studies involving human embryonic stem cell-derived RPE (hESC-RPE) and induced pluripotent stem cell-derived RPE (iPSC-RPE) have yielded promising results (Table 2). U.S.-based Advanced Cell Technology (now Ocata Therapeutics) and Japan’s RIKEN Center have focused on STGD, demonstrating that subretinal delivery of RPE cells is feasible, well-tolerated, and associated with encouraging anatomical improvements, thereby supporting further clinical development [7, 22, 33, 39]. Additionally, MSC-based therapies have been evaluated for RP and STGD, with intravitreal, subtenon, and suprachoroidal injections yielding positive outcomes [33, 39]. The source of MSCs and the delivery route, particularly the suprachoroidal injection of umbilical cord-derived MSCs, emerged as key factors influencing therapeutic efficacy [38, 40]. Adverse events were primarily localized ocular issues with minimal systemic effects, further supporting the safety of these approaches [22, 30, 33].

### Regulatory, ethical, and technical challenges

The U.S. Food and Drug Administration (FDA), European Medicines Agency (EMA), Central Drugs Standard Control Organization (CDSCO), and Pharmaceuticals and Medical Devices Agency (PMDA) are among the regulatory bodies responsible for monitoring treatments for IRDs. These organizations mandate comprehensive clinical trials, adherence to Good Manufacturing Practices (GMP), and complete cell traceability to ensure safety and effectiveness [5, 11, 46]. Ethical concerns related to embryonic stem cells (ESCs) include issues of informed consent, particularly for disadvantaged populations, and the risks associated with unlicensed stem cell clinics offering unproven therapies [8, 9]. Access to these therapies is further restricted by the high costs associated with stem cell manufacturing, storage, and testing, particularly in low-income regions, thereby exacerbating healthcare inequities [6, 46]. Unresolved technical challenges remain regarding graft rejection, immunological incompatibility, post-transplant cell survival, large-scale expansion, and cell purification [2, 5, 8]. Furthermore, logistical barriers and the absence of standardized surgical techniques hinder widespread clinical adoption. However, innovative solutions, such as CRISPR/Cas9 gene editing, 3D bioprinting for retinal restoration, and AI-driven therapeutic optimization, may provide pathways to address these challenges. Genetic profiling for personalized treatments may also enhance therapeutic efficacy and equity in the care of IRDs.

### Future directions and barriers to clinical adoption

Stem cell therapy for IRDs faces several challenges before achieving routine clinical use. Key obstacles include high manufacturing costs and the lack of standardized protocols for cell differentiation, preparation, and delivery. The risk of immune rejection and significant variations in regulatory requirements across regions also pose major concerns. The proliferation of unregulated clinics providing unproven interventions undermines patient confidence and hampers responsible progress in this field. Advancements depend on adherence to GMP-level manufacturing, continuous functional evaluations, patient-reported outcomes, and improved coordination among regulatory systems. Ongoing research is focusing on allogeneic iPSC-RPE and MSC preparations, as well as strategies utilizing biomaterials to support graft survival and approaches combining gene repair with cell-based replacement. The realization of the therapeutic potential of stem cell-based strategies will hinge on rigorous clinical validation, standardized procedures, and the prioritization of patient-centered outcomes.

## Conclusion

IRDs lead to progressive and irreversible vision loss, with no widely available curative treatments. Early human studies utilizing MSCs, RPCs, or pluripotent-derived RPE cells indicate that these therapies can be safely administered via intravitreal, subretinal, or suprachoroidal routes, even in advanced stages of the disease. Some participants have demonstrated transient improvements in visual function, as measured by best-corrected visual acuity (BCVA), visual fields, or electrophysiological metrics; however, these effects remain uncertain, variable, and non-reproducible. Most evidence is derived from small, early-phase trials lacking control groups, with variability in cell sources, preparation methods, delivery techniques, and outcome measures, thereby limiting interpretability. Proposed mechanisms, including paracrine signaling, immunomodulation, or limited cell integration, may contribute to neuroprotection; however, permanent retinal cell replacement or functional restoration has yet to be achieved. Consequently, large, well-controlled trials with extended follow-up periods are necessary to ascertain the therapeutic potential of these interventions for IRDs.
